# A Parallel Mediation Analysis on the Effects of Pandemic Accentuated Occupational Stress on Hospitality Industry Staff Turnover Intentions in COVID-19 Context

**DOI:** 10.3390/ijerph191912050

**Published:** 2022-09-23

**Authors:** Lavinia Denisia Cuc, Andrea Feher, Paul Nichita Cuc, Silviu Gabriel Szentesi, Dana Rad, Gavril Rad, Mioara Florina Pantea, Cosmin Silviu Raul Joldes

**Affiliations:** 1 Faculty of Economical Sciences, Aurel Vlaicu University of Arad, 310032 Arad, Romania; 2 Department of Economy and Firm Financing, University of Life Sciences “King Mihai I” from Timisoara, 300645 Timisoara, Romania; 3 Department of Statistical Science, University College London, London WC1E 6BT, UK; 4 Center of Research Development and Innovation in Psychology, Aurel Vlaicu University of Arad, 310032 Arad, Romania; 5 Faculty of International Business and Economics, Bucharest University of Economic Studies, 010374 București, Romania

**Keywords:** hospitality industry, turnover intentions, pandemic accentuated occupational stress, occupational safety and health perception, perceived organizational effectiveness, perceived job insecurity, parallel mediation analysis

## Abstract

The purpose of this research was to analyze how different effects of the COVID pandemic, expressed through pandemic accentuated occupational stress, perceived job insecurity, occupational safety and health perception and perceived organizational effectiveness, may impact turnover intentions of the personnel in the hospitality industry. Our research team designed an online questionnaire which was analyzed with network analysis to depict the relationship between factors, and, then, a confirmatory factor analysis was employed to confirm the distribution of the items to the envisaged five factors. Based on a sample of 324 randomized Romanian hospitality industry staff, the results of our cross-sectional study revealed that occupational safety and health perception, perceived organizational effectiveness and perceived job insecurity in the pandemic accentuated occupational stress to indirectly and significantly impact hospitality industry staff turnover intentions (TI). The results indicated that, while the total effect of PAOS on TI was significant, the direct effect was still significant, while all three mediators remained significant predictors. Overall, mediators partially mediated the relationship between PAOS and TI, indicating that employees with low scores on occupational safety and health perception (OSHP), and perceived organizational effectiveness (POE) and high scores on perceived job insecurity (PJI) were more likely to have higher levels of TI turnover intentions.

## 1. Introduction

The hospitality industry is a substantial subset of the service industry and is divided into four major categories: food and beverage, travel and tourism, hotels, and leisure [1,2].

Personnel turnover, as a result of turnover intentions, is a common issue and a significant burden for employers globally in the hospitality business [3,4,5,6,7].

A substantial amount of research indicates that efficient management of workplace challenges employing positive psychological abilities of personnel might reduce turnover intentions [8,9,10,11,12,13,14,15,16].

The hospitality industry’s hotels and restaurants had the highest drop in personnel numbers, while, even before the start of the epidemic, around 65% of tourist enterprises reported having difficulty in paying their obligations and cited financial constraints [17].

As a result of the crisis, millions of individuals in the hospitality industry have lost their employment and fallen into poverty, while others have experienced extraordinarily high levels of job instability and the psychological and physical consequences that come with that [18].

The pandemic context has dramatically and irreversibly changed how research and practice approach the turnover intentions appraisal, especially in the hotel industry. If pre-pandemic research was more focused towards enhancing job conditions and well-being of employees [19,20], current research tends to be more focused on “survival skills”, and how to lower the negative psychological impact and mental and physical health related problems that have emerged from the precariousness of job opportunities in this business.

In a recent study [21], the authors discovered that work insecurity and organizational changes were negative predictors of job motivation and job satisfaction, while risk-taking behavior was simply a negative predictor of job satisfaction. As control variables, the relevance of demographic features revealed that age and marital status had a substantial influence on job motivation and turnover intentions.

Employee turnover has been noted by researchers [22,23,24,25] as a major and demanding challenge for organizations in the hospitality sector, also from the career construction theory perspective, emphasizing the need to know the variables that lead to high turnover rates in this industry in order to assist in tackling the issue.

Previous research in the hospitality sector has found several predictors of personnel turnover intentions, including job satisfaction [26], work–family conflict [27], organizational justice [28], psychological contract [29], and affective commitment [30]. However, there has been little study on the importance of career by flexibility as a predictor of turnover intentions in the hospitality business [21]. Research [21] indicated that happiness orientation is a relevant component, agreeing that when perceived career opportunities [31,32,33] are minimal, employees are more likely to consider alternative career options beyond their companies, even when career plasticity leads towards happiness orientation.

This research presents the results of our examining the role of occupational safety and health perception, perceived organizational effectiveness, and perceived job insecurity in pandemic accentuated occupational stress and how the hospitality industry may shape employee turnover intentions. By analyzing the parallel mediation role of occupational safety and health perception, perceived organizational effectiveness and perceived job insecurity, we assume that pandemic accentuated occupational stress can be tackled in order to lower hospitality industry staff turnover intentions in the COVID-19 context. In light of this research, it is argued that a positive perception of occupational safety and health, positively perceived organizational effectiveness and a low perception regarding job insecurity, or positive perception of job security, significantly and positively impacts hospitality industry staff turnover intentions.

### 1.1. Psychological Factors Influencing Turnover Intention in Hospitality Industry in the Context of COVID

Personnel turnover has long been a key management issue in the hotel industry, having both direct and indirect costs, including the price of hiring and training new staff, as well as the loss of organizational expertise and culture [34]. In order to appreciate the phenomenon and its ramifications for the hospitality business, much focus has been placed on worker turnover intention [35,36,37].

We began by looking at the comprehensive study in [35], which found convincing evidence regarding the effects of work attitudes, burnout, and role stressor conflicts on turnover intention. From there, we further looked into which psychological factors had the greatest effects on employees’ intentions to leave their jobs in the Romanian hospitality sector.

In a different meta-analysis [38], it was shown that in the hospitality sector, there is a relatively negative correlation between organizational commitment and employee turnover intention. Individuals who have an emotional connection to their workplaces are less likely to plan to leave. In the hotel sector, talent management, performance assessment methods, promotions, prospects for training and development, career options, and talent management not only increase corporate commitment but also attract and retain talented personnel.

Recent research [39], efficiently presented evidence that career flexibility is connected to hospitality employee turnover intentions, using career construction theory (CCT). According to the study findings, career flexibility is inversely connected to employee turnover intentions via orientation to happiness, implying that orientation to happiness is an underlying factor. Furthermore, perceived job potential was shown to be a key boundary condition, with lower levels of perceived career opportunity resulting in a weaker mediated association.

Another research [40], looked at how psychological capital affected how stressed hotel workers were about their jobs and how likely they were to leave. It has been demonstrated that psychological capital and job stress have a significant impact on turnover intentions. The relationship between job stress and intentions to leave was mediated by employees’ psychological capital, or optimism. Employed staff at five-star hotels are also more stressed, whereas employees in four-star hotels are more optimistic and resilient. In terms of management, middle management is more stressed, whereas top management is more efficacious, hopeful, optimistic, and resilient. The interaction effect demonstrates that middle management personnel at three-star hotels have high turnover intentions, but employees in five-star hotels have reduced turnover intentions, and vice versa for top management. This study has significance in the hospitality business for strengthening stress management approaches, which may aid in lowering turnover intentions.

According to the findings [41], the following six characteristics were statistically significant in predicting employee turnover in the hospitality industry: job instability, job discontent, lack of organizational commitment, bad working conditions, improved employment possibilities, job stress, and unjust treatment. Management should be dedicated to building smart and successful retention tactics by offering appropriate pay policies, better working conditions, strong communication channels between management and staff, and chances for training and growth. They should also build a feedback system to learn what employees think about their employment, their attitudes about their professions, what drives them to perform well, and what kind of organizational practices demoralize and, finally, drive them to leave the business.

Thus, past research found a substantial positive link between job satisfaction and workload and income, as well as company assistance. Job happiness was unaffected by coworker relationships. Furthermore, employee turnover intentions were significantly influenced by work satisfaction. Workload and compensation were the most influential factors in deciding whether or not to exit the hospitality industry.

### 1.2. Pandemic Accentuated Occupational Stress

The COVID-19 pandemic has put service providers across all sectors under extreme pressure. Concerns about workplace stress have become more common, including shared trauma and burnout. Although these issues are not novel to the hospitality sector, the COVID pandemic has brought attention to the importance of equipping staff with the most practical and efficient methods for lowering stress and burnout. The limitations of self-care, which is generally praised as a basis for lowering shared stress and burnout, are also highlighted by the COVID pandemic. Although self-care can be helpful in some occupations, the organization should assume the majority of the burden for lowering burnout and shared trauma [42].

The findings indicate that how individuals perceive the demands of their employment influences the strength and direction of the links between various types of job demands and employee outcomes. Furthermore, the findings suggest that perceived organizational support has a mediation role in lowering the negative impacts of work demands on employee outcomes. According to one study, positive affectivity attenuated the link between job intensification and employee burnout [43].

Work-related stress had a negative and significant influence on work–life balance, a positive and significant impact on time stress, and a negative and significant impact on anxiety, according to the findings [44].

Empirical research emphasizes the need to care for employees since they are concerned about their own health and the health of their families, as well as being terrified of losing their jobs. A variety of characteristics that influence employees’ well-being, motivation, safety practices, and intentions to leave during this crisis have also been found. Hotel managers are advised on how to efficiently manage human resources during a pandemic, such as building a safety culture and fostering open communication [45].

### 1.3. Staff Turnover Intentions in COVID-19 Context

An employee’s desire to leave their current employer permanently at some time in the near future is referred to as their own projected likelihood of doing so [46].

Recent research has shown that COVID-19 negatively impacted employee engagement in premium hotels due to feelings of job instability. Employees with low levels of job engagement, zeal, and dedication are more likely to consider leaving their jobs, and job instability brought on by the COVID crisis is a key contributor in lowering workplace engagement [47].

Employee turnover has been a difficulty for hotel corporations, who have traditionally expressed concern about delivering excellent service to their customers [48]. In both developed and emerging regions, the hotel business is confronting labor shortages [48,49,50]. Employee turnover has become the most difficult managing concern since it is consistently higher in the hotel industry than in other sectors [51,52].

Furthermore, COVID-19 posed significant issues in staff recruitment and retention, emphasizing the significance of hotel employee turnover intentions during this pandemic [53]. Furthermore, hotel staff may depart due to a perceived insecure situation caused by the severe COVID-19 operational environment [54,55,56]. As a result, understanding the causes of staff turnover difficulties in the hospitality business during the COVID-19 pandemic is critical.

A meta-analysis found a substantial and consistent link between turnover intent and actual turnover rate, so turnover intent can be seen as a major antecedent to the actual rate of voluntary turnover [57,58]. The variable has, therefore, often been employed in prior research [59] since it is far simpler to communicate the intention to leave a job than it is to actually do so, and the current study similarly included turnover intent as a dependent variable.

Many studies have found that higher engagement induces lower turnover intent and that engagement is the most influential psychological variable in reducing the turnover of employees [60,61,62].

### 1.4. Occupational Safety and Health Perception

According to a European poll on working conditions, one in three European workers fear that their health and safety are in danger due to their jobs [63]. As a result, risk perceptions are impacted by hazardous working circumstances and the likelihood of contracting occupational illnesses and having accidents.

According to the study on the creation of a safety and health management system at work [64], the management of the company should prioritize ensuring the best possible working conditions and safety in order to reap financial and organizational benefits, such as lowering costs of any kind, enhancing the organization’s reputation in the eyes of competitors, and boosting productivity.

The findings of two case studies demonstrate that the hospitality industry may be stressful, and that many employees are vulnerable because of unfavorable working circumstances and poor wages. A high prevalence of casualization and workforce turnover, as well as low trade union presence, were found by [65]. In response to the COVID crisis, a study was conducted to examine the opinions, experiences, and future prospects of occupational safety and health professionals (OSHPs) [66]. The analogy implied that the OSHP’s mandate would be expanded to cover business continuity, resilience, and welfare in addition to identifying and reducing risks brought about by the pandemic. Employees’ perceptions of safety and health climates during the first wave of COVID were closely related to their self-reported well-being. Employee well-being may be impacted by safety and health climates even when other disruptions occur, therefore enterprises with strong climates may be better equipped to preserve it during calamities [67].

### 1.5. Perceived Organizational Effectiveness

The realization that employee security and safety are key indices of the company indicates a positive perception of the economic and organizational benefits. An optimal organizational approach involves ensuring a safe and healthy workforce where occupational safety is prioritized, and harmful and hazardous factors are kept to a minimum [64]. The main resource of every company is represented by its employees, who are of particular importance as they create the company’s production, but are, also, an expensive resource. Managing employee behavior and motivating their work should become a priority. Company management should create conditions where employees feel satisfied with their work in the organization. Workplace health and safety are requirements for boosting the efficiency of corporate activities [64].

Stress and perceived organizational effectiveness have a negative relationship. The findings imply that the kind of stress influences the link between stress and production. In all four firms, the most prevalent kind of stress was dysfunctional stress. Furthermore, dysfunctional stress levels explained more variance in effectiveness levels than overall stress levels [68].

A strong predictor of willingness to resign was the association between organizational commitment and perceived support. Employees who are less committed but get good organizational support are less likely to leave their jobs. This provides a strategy for managers who are trying to keep valuable employees whose dedication alone might not be enough to keep them because perceived organizational support was found to affect turnover [69].

Employee perceptions of the amount of funding provided to the agency affected how well the organization was doing. Additionally, it was found that critical factors were employee job performance, levels of enthusiasm for public service, the degree of position ambiguity and employee participation in decision-making [70].

### 1.6. Perceived Job Insecurity

Through their effects on psychological wellbeing, insecure work behaviors are indirectly associated with turnover intentions and risk behavior [71]. Organizational commitment mediated the connection between work insecurity and turnover intention, which was positively correlated with job insecurity in [72].

In all countries, perceived employment insecurity increases intentions to change jobs, according to the research. Additionally, in most nations, perceived employability raises turnover intentions, although there is less data that suggests that workers who believe they are irreplaceable are less likely to have such intentions. A general conclusion on buffering effects cannot be drawn since the data on how employees who perceive their employability or irreplaceability respond to job insecurity, in terms of their intentions to leave, varied significantly between countries [73].

Nonetheless, a recent study found that job instability, desire to leave, and occupational well-being are, to some extent, comparable experiences across work departments. Two ideas impacted this research: resource conservation and emotional contagion theories. Poor occupational well-being mediated the relationship between job insecurity and intention to leave, and cross-level interactions revealed that the greater the negative impact of job insecurity on individual well-being, the lower the level of well-being at the work department level. As a result, if the employee is unsatisfied at the work department level, the negative relationship between job instability and individual well-being may be exacerbated [74].

On the one hand, job instability stimulates performance, while on the other, it has a detrimental impact on personnel attitudes or views at work. The job’s normal operating procedures offer one rationale for perceived employment instability. Significant implications for change management in organizations have been found through job insecurity and teamwork or workplace friendship in today’s changing environments, becoming a motivating factor for team performance [75].

The negative impacts of employing job uncertainty as a motivating tool to boost employee performance should be taken into account by company management [76]. Job uncertainty has an impact on both the physical and emotional well-being of employees [77]. The most crucial tactics in the connection between job instability and mental health relate to social contact both inside and outside of an organization. As a result, developing strong social networks at the workplace is a guarantee of wellbeing [78].

## 2. Materials and Methods

### 2.1. Participants

In terms of sociodemographic characteristics, out of a total of 324 participants, 38% represented masculine respondents and 62% represented feminine respondents. In terms of age span, 6% declared themselves to be between 18 and 20 years old, 33% between 21 and 30 years old, 32% between 31 and 40 years old, and 29% between 41 and 65 years old.

In regards to their educational background, 1% declared elementary studies, another 1% declaredd gymnasia studies, 8% declare professional studies, 40% declared high-school studies and 50% declared higher education studies. As for previous work experience, 14% declared under 1 year, 30% declared between 1 and 3 years, 20% declared between 3 and 5 years, 16% declared between 5 and 10 years and 20% declared more than 10 years of professional experience.

In terms of monthly income, 5% declared a monthly income of under 1500 ron, 43% declared between 1500 and 2500 ron, 38% declared between 2500 and 5000 ron, 9% declaredd between 5000 and 7500 ron and 5% declared above 7500 ron. Their jobs ranged from unqualified work to managerial positions.

In terms of turnover intentions, this questionnaire used a single item question: “If I had the chance to change jobs, I would without reservation”. The following answers were registered: 46% completely disagreed, 12% disagreed, 19% were neutral, 11% agreed and 12% completely agreed.

### 2.2. Procedure

We followed the recommended phases in [79] to create the following valid and reliable scale: (1) item development (domain identification, item generation, and content validity); (2) scale development (question pretesting, sampling and survey administration, item reduction, and factor extraction); (3) scale evaluation (tests of dimensionality, reliability, and validity).

Firstly, we analyzed existing theories on turnover intention, workplace safety and health perception, COVID fear, pandemic accentuated occupational stress, perceived organizational effectiveness, and job insecurity in the context of the hotel sector during the COVID crisis.

Then, our team analyzed items from the available literature to create 16 items that corresponded to the aforementioned categories.

For turnover intentions we opted to use a single item measure. In scientific literature, the single item measurement of turnover intentions has proven to be a valid and reliable measurement, with studies covering the years 1982–2020 [80,81,82,83,84,85,86]. The other 15 items referred to the following: occupational safety and health perception (5 items), which were inspired by [87], COVID fear (3 items), that were adapted from [88], pandemic accentuated occupational stress (3 items), that were adapted from [89], perceived organizational effectiveness (2 items), that were inspired by [90] and perceived job insecurity (2 items), inspired by [91]. We opted to design a new scale based on scientific literature descriptors for each studied dimension, mainly due to seeking proper adaptation for the COVID context.

We then sent the 16-item questionnaire to a panel of 5 experts in economics, psychology, and sociology and asked them to score the relevance of each item on a Likert scale from 1 to 5, and then determined Cohen’s coefficient kappa (k). For all items, the k values indicated rater agreements were in the ideal range (0.90). Then, we emailed our questionnaire to a sample of 10 respondents (the previous year’s Psychology students) and asked them to assess each item on a Likert scale of 1 to 5, indicating their comprehension. Then, using a cognitive interview, we evaluated pilot respondents’ opinions on each item. All items were validated; therefore, we sent our questionnaire to the main participants in the Romanian hospitality industry through email. A total of 324 replies were received in the questionnaire google form’s excel database.

### 2.3. Measurement

Our 16 items questionnaire, designed for this research, was purposely structured on the following dimensions: turnover intentions (1 item), occupational safety and health perception (5 items), COVID fear (3 items), pandemic accentuated occupational stress (3 items), perceived organizational effectiveness (2 items) and perceived job insecurity (2 items).

According to our methodology, the following steps were followed: tests of dimensionality, tests of reliability, and tests of validity [79].

**Figure 1:**
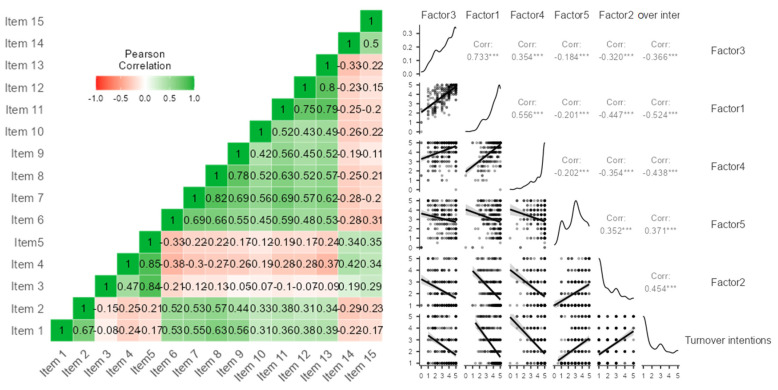
Correlations between the scale items, the 5 factors and turnover intentions. *** significant at *p* < 0.001.

Before running the confirmatory factor analysis (Jamovi software, https://www.jamovi.org, accessed on 16 August 2022), we analyzed (Figure 1 and Table 1) the correlations between the 5 scales and turnover intentions. The results showed significant positive correlations between turnover intention (TI) and Factor 2, Pandemic accentuated occupational stress (PAOS) and Factor 5, Perceived job insecurity (PJI). There were significant negative correlations between TI and Factor 3, COVID fear, Factor 1, Occupational safety and health perception (OSHP) and Factor 4, Perceived organizational effectiveness (POE). Correlations spanned between −0.524 and 0.733 and were all significant at *p* < 0.001.

As we expected, the higher the turnover intention was (m 2.28), the higher were pandemic accentuated occupational stress (m 2.08), and perceived job insecurity (m 2.98), and the lower were COVID fear (m 3.55), satisfaction over the occupational safety and health issues (m 4.03) and the perceived organizational effectiveness (m 4.25).

In terms of reliability, our scale presented a Cronbach’s α of 0.729, with a grand mean of 3.62, and a standard deviation of 0.56.

**Figure 2:**
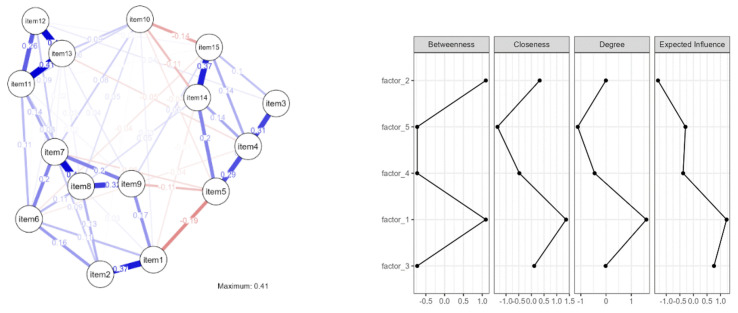
Network analysis and centrality plot for the 5 factors of the questionnaire.

Next, we performed a network analysis (Jasp software, Amsterdam, The Netherlands) of the 16 items to deeper understand the relationship between them and to verify the structure of the 5 presumed factors (Figure 2 and Table 2), from a behavioral economics perspective. Behavioral economics blends economics and psychology to better understand how and why individuals act as they do in the real world [92,93]. Recent empirical and theoretical assessments of social networks were reviewed, with an emphasis on how social networks affected economic behavior and how social networks arose.

As seen in Figure 2 and Table 2, there were four centrality measures employed to identify highly influential nodes: betweenness, closeness, strength and expected influence [94].

The centrality of closeness reflects how near a node is to all other nodes in the network. It is determined as the average of the shortest path lengths between each node in the network. The greater a node’s centrality, the shorter its total distance to all other nodes. Closeness may be thought of as a measure of how long it takes to disseminate information sequentially from one node to all other nodes. The number of times a node is on the shortest path between other nodes is measured by betweenness centrality. Betweenness centrality is commonly seen as a measure of other nodes’ reliance on a certain node, and, therefore, of potential control. The strength of a node is the total of the absolute value of its connections with other nodes in the network, and it is used to evaluate a node’s impact with its immediate neighbors, or nodes with which it has an edge [94].

Item 5 had the biggest effect over the flow between all items in terms of betweenness. “The lack of inadequacy of security measures adopted by the Company to prevent the transmission and contamination of COVID causes me significant professional stress”, explained why Factor 2, pandemic accentuated occupational stress, had the most impact over the other variables.

In terms of closeness, the item best placed to influence the entire network most quickly was item 9. “The company follows the new norms and procedures created to combat and guard against COVID, which have also been required of the personnel” described and reinforced the power of Factor 5, occupational safety and health perception, had over the entire network.

In terms of strength, the most influential item over its immediate neighbors was item 7. “I think that the precautions adopted in the hotel/restaurant where I work have totally safeguarded me against COVID infection” once again confirmed the contamination effect of Factor 2, pandemic accentuated occupational stress, over the other factors.

In terms of expected influence, item 8, “I am completely confident that the anti-COVID procedures implemented by the firm where I work effectively safeguard the clients” from Factor 1, occupational safety and health perception, presented the most prominent characteristics in the analyzed network.

Factor 3, COVID fear, and Factor 4, perceived organizational effectiveness, contributed, but had marginal effects.

After looking at the influential potential that items and factors had, we employed a confirmatory factor analysis to verify the distribution of items across predicted factors. The minimum residual extraction method was used in combination with an ‘oblimin’ rotation, and a forced model of 5 factors.

As seen in Table 3, all factor loadings ranged between 0.449 and 1.041, confirming the distribution of all items to the envisaged theoretical factors.

Factor 1 (Occupational safety and health perception) (OSHP) was composed of the following items: Item 6, “*I am pleased with the new workplace policies and procedures, and I do not sense any threat or insecurity as a result of the COVID infection*.”; Item 7, “*I think that the precautions adopted in the hotel/restaurant where I work have totally safeguarded me against COVID infection*.”; Item 8, “*I am completely confident that the anti-COVID procedures implemented by the firm where I work effectively safeguard the clients*.”; Item 9, “*The company follows the new norms and procedures created to combat and guard against COVID, which have also been required of the personnel*.”; and Item 10, “*I have zero concerns about getting to work safely or running the risk of contracting the COVID infection*”.

Factor 2 (Pandemic accentuated occupational stress) (PAOS) was composed of the following items: Item 3, “*I am particularly concerned about the security and labor protection standards set in relation to COVID protection*.”; Item 4, “*I am intellectually and emotionally fatigued as a result of the worry caused by the possibility of infection with COVID*.”; and Item 5, “*The lack of, or inadequacy of, security measures adopted by the Company to prevent the transmission and contamination of the COVID causes me significant professional stress*”.

Factor 3 (COVID fear) (CF) was composed of the following items: Item 11, “*I am not concerned about the prospect of contracting COVID at work and spreading the virus on to my family*.”; Item 12, “*I am not concerned about becoming infected with COVID by clients at my workplace.*”; and Item 13, “*I am not concerned about becoming infected with COVID by coworkers*”.

Factor 4 (Perceived organizational effectiveness) (POE) was composed of the following items: Item 1, “*I appreciate that, in the midst of the present pandemic, the firm (Hotel) where I work has adopted adequate and necessary preventive measures against the spread of COVID*.”; and Item 2, “*I appreciate that, in the present pandemic crisis, the firm (Hotel) where I work has made appropriate and necessary efforts to maintain employment that may be affected by the pandemic’s economic-financial position*”.

Factor 5 (Perceived job insecurity) (PJI) was composed of the following items: Item 14, “*I am concerned about the long-term viability of my job*.”; and Item 15, “*Because of the pandemic circumstances, I am concerned about my employer’s financial viability*”.

To measure turnover intentions, we used Item 16, “*If I had the chance to change jobs, I would do it without reservations*”, that was not included in the confirmatory factor analysis.

According to the factor loadings summary, the Factor 1 explained 21.66% of the variance, the Factor 2 explained 14.89% of the variance, Factor 3 explained 15.84% of the variance, Factor 4 explained 9.37% of the variance, and Factor 5 explained 8.28% of the variance, with a total variance of 70%, a very high percentage.

The KMO measure of sample adequacy [95] yielded an MSA coefficient between 0.420 and 0.963 and Bartlett’s Test of Sphericity indicated a χ^2^ of 3486 at df (105) and a *p* < 0.001.

As for model fit measures, the values obtained for RMSEA (0.168) and TLI (0.735) did not fit within acceptable limits [96], due to inappropriately incorporating the measurement model as a factor model.

Since it was not the purpose of this paper to validate the scale, only to get reliable short measures for our five theoretical factors, we further proceeded with testing our hypothesis that presumed a parallel mediation of occupational safety and health perception (OSHP), perceived organizational effectiveness (POE) and perceived job insecurity (PJI) over the relationship between pandemic accentuated occupational stress (PAOS) and hospitality industry staff turnover intentions (TI).

## 3. Results

Before running the parallel mediation analysis (SPSS V.26 PROCESS MACRO software, IBM Corp: Armonk, NY, USA), we first wanted to check if all 5 factors, OSHP, POE PJI, PAOS and CF, explained a significant amount of variance in TI, further indicating a parallel mediation situation.

The linear regression analysis revealed that 36% variance of TI was explained by OSHP, POE, PJI, PAOS and CF, with an F of 36.9 (5, 310) at *p* < 0.001. Generally, a VIF above 4 or tolerance below 0.25 indicates that multicollinearity might exist, but in our case all VIF were below 2.9 and tolerance values above 0.34, with a Durbin–Watson coefficient of 0.687 at *p* < 0.001 and Shapiro-Wilk coefficient of 0.958 at *p* < 0.001.

The regression analysis coefficients revealed that OSHP (F = 14.307, *p* < 0.001), POE (F = 8.984, *p* < 0.003), PJI (F = 12.288, *p* < 0.001), and PAOS (F = 14.578, *p* < 0.001) were all predicting factors of TI, except for CF (F = 0.115, *p* = 0.734). Table 4 presents the beta coefficients for all factors. Except for CF, OSHP and PJI negatively impacted TI while PAOS and POE positively impacted TI.

The higher the occupational safety and health perception and perceived organizational effectiveness were, the lower was the turnover intention of Romanian hospitality industry staff, while the higher the pandemic accentuated occupational stress and perceived job insecurity were, the higher was the turnover intention of Romanian hospitality industry staff.

Lastly, we opted to use the Process Model 4 [97], which calculates a parallel mediation model where the indirect effect of PAOS over TI is mediated by three mediators parallel to each other: OSHP, POE PJI.

Step-by-step results from the parallel mediation analysis revealed that *a* path for the first mediator, Factor 1, OSHP, obtained a regression value of −0.3439 at *p* < 0.001, demonstrating the significantly negative effect of the independent variable, PAOS, on mediator 1, Factor 1, OSHP. Then, *a* path for the second mediator, Factor 4, POE, obtained a regression value of −0.2698 at *p* < 0.001, demonstrating the significantly negative effect of the independent variable, PAOS, on the mediator 2, Factor 4, POE. The last *a* path for the third mediator, Factor 5, PJI, obtained a regression value of 0.3388 at *p* < 0.001, demonstrating the significantly positive effect of the independent variable PAOS on the mediator 3, Factor 5, PJI.

We further present the coefficients obtained for all *b* paths for the three mediators. The *b* path for the first mediator, Factor 1, OSHP, obtained a regression value of −0.4248 at *p* < 0.001, demonstrating the significantly negative effect of mediator 3, Factor 1, OSHP, on the dependent variable TI. The *b* path for the second mediator, Factor 4, POE, obtained a regression value of −0.2404 at *p* < 0.001, demonstrating the significantly negative effect of the mediator 3, Factor 4, POE, on the dependent variable TI. Finally, the *b* path for the third mediator, Factor 5, PJI, obtained a regression value of 0.1944 at *p* < 0.001, demonstrating the significantly positive effect of the mediator 3, Factor 5, PJI, on the dependent variable TI. The direct effect *c* prime was still significant for the independent variable, Factor 2, PAOS, and the regression value of 0.2232 at *p* < 0.001 was still a significant predictor of the dependent variable, TI; both results validated the parallel mediation hypothesis. The total effect of the PAOS on the TI was 0.5000 at *p* < 0.001, with LLCI of 0.3910 and ULCI of 0.6089. The direct effect of the PAOS on the TI was 0.2232 at *p* < 0.001, with LLCI of 0.1084 and ULCI of 0.3379.

The level of confidence for all confidence intervals in output was 95.0000 and the number of bootstrap samples for percentile bootstrap confidence intervals was 5000.

**Figure 3:**
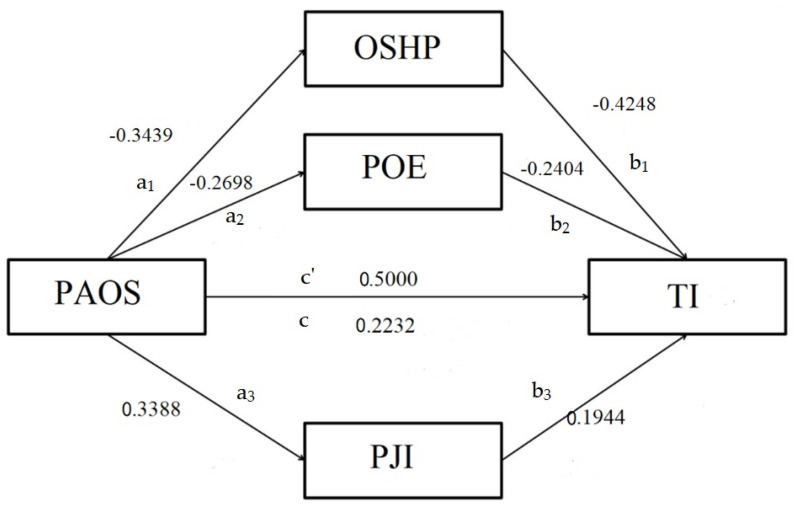
Parallel mediation analysis comparing the indirect effects of OSHP, POE and PJI. N = 324 The a, b and c paths represent regression coefficients that are all significant in terms of 95% bias-corrected confidence intervals that do not contain zero (5000 bootstrapped samples). c = total effect, c′ = direct effect (when a and b are accounted for).

The three mediator variables were analyzed simultaneously while controlling for the effect of one another [98]. The results based on the 5000 bootstrapped samples indicated that, while the total effect of PAOS on TI was significant (βtotal = 0.5000, SE = 0.0554, *p* < 0.001), the direct effect (βdirect = 0.2232, SE = 0.0583, *p* = 0.2576) was still significant, while all 3 mediators remained significant predictors (Figure 3).

Overall, the three mediators partially mediated the relationship between PAOS and TI (IE overall = 0.2768, SE = 0.0426, 95% CI: LL = 0.2010 to UL = 0.3684), indicating that employees with low scores on occupational safety and health perception OSHP and perceived organizational effectiveness POE, and high scores on perceived job insecurity PJI, were more likely to have higher levels of TI turnover intentions. 

## 4. Discussion

Overall, our findings were consistent with the existing relevant scientific literature.

The negative link between perceptions of workplace health and safety and pandemic-exacerbated occupational stress was comparable with the findings of [99], which indicated a high association between perceived dangers related to the presence of COVID pandemic and psychological distress.

The findings that job stress had a significant positive relationship with employees’ turnover intentions, whereas perceived organizational support had a significant negative relationship with employees’ turnover intentions, and that perceived organizational support also hindered the positive relationship between job stress and personnel’ turnover intentions, were consistent with the finding that perceived organizational support had a significant negative relationship with employees’ turnover intentions [100].

The strong link between pandemic accentuated occupational stress and level of job insecurity felt was similar to the findings of [101]. Individual views of work insecurity and job stress were found to have a beneficial influence on job stress, and psychological capital considerably lowered perceptions of job insecurity and job stress. The authors in [102] discovered a positive relationship between job uncertainty and work stress. Another related research found a substantial relationship between COVID perceptions, work insecurity, and psychological characteristics. Greater levels of COVID perceptions were associated with higher degrees of job insecurity, anxiety, depression, job burnout, and job alienation in [103].

We uncovered substantial supporting evidence in the current literature indicating a negative relationship between occupational health and safety perceptions and perceived organizational performance and turnover intentions. The authors in [104] revealed a negative association between workplace health and safety and desire to leave, as well as a positive relationship between occupational health and safety and organizational commitment. Employee satisfaction with their organization’s health and safety system was more likely to engender commitment and lead to low turnover intention, and organizational commitment significantly mediated the relationship between occupational health and safety and turnover intention.

As for the positive correlations between perceived job insecurity and pandemic accentuated occupational stress with turnover intentions, there was also strong supporting evidence in the existing literature. According to a recent study [105], three role stressors and job instability positively increased job stress, which led to turnover intentions. The work in [106] also achieved comparable findings. The results revealed that organizational support and commitment had a lowering influence on personnel desire to leave, and that effects varied dramatically depending on individuals’ feelings of being infected by COVID and job insecurity.

As a consequence, we propose that, within the framework of social exchange theory, the favorable support staff in the hospitality sector get from their employers boost the company’s image. In [107] it was contended that, in the framework of social exchange theory, organizational support and implicitly perceived organizational success provided by organizations to staff impacted employee behavior. Business organizational support for employees becomes increasingly important during stressful times, such as pandemics. We believe that providing assistance to hotel employees affected by the pandemic, as well as decreasing turnover intentions and improving organizational engagement, would result in long-term benefits.

## 5. Implications

According to social exchange theory, on which this study was grounded, social conduct is the product of an exchange process. The goal of this transaction is to maximize advantages while minimizing expenditures. Individuals consider the possible rewards and hazards of their social ties, according to this hypothesis. They cancel or abandon the partnership if the risks outweigh the advantages [108].

The importance of reciprocal exchange interactions between organizations and their employees is emphasized in key lines of management research. When employees believe they are supported by their employers, they engage with increased work motivation [109].

This study developed a conceptual model for the hotel sector based on employee–organization interaction using social exchange theory as a theoretical foundation. The purpose of this research was to consider the effects of pandemic accentuated occupational stress on hospitality industry staff turnover intentions in the COVID context, based on three parallel mediators: occupational safety and health perception, perceived organizational effectiveness and perceived job insecurity.

Pandemic accentuated occupational stress had a significant relationship with occupational safety and health perception, perceived organizational effectiveness and perceived job insecurity, which also had a significant relationship with hospitality industry staff turnover intentions. Occupational safety and health perception, perceived organizational effectiveness and perceived job insecurity partially mediated the relationship between pandemic accentuated occupational stress and hospitality industry staff turnover intentions.

Although perceptions of occupational safety and health, perceived organizational effectiveness, and perceived job insecurity are being studied more in the hospitality profession, there is minimal information available about their antecedents and results. As a result, this study builds on earlier research by using social exchange theory to address gaps in the existing literature and present a conceptual framework for the hotel industry based on employee-organization interaction.

## 6. Conclusions

Based on a sample of 324 randomized Romanian hospitality industry staff, the results of our cross-sectional study revealed that occupational safety and health perception, perceived organizational effectiveness and perceived job insecurity in pandemic accentuated occupational stress indirectly and significantly impact hospitality industry staff turnover intentions. Based on the results of 5000 bootstrapped samples, the total effect of PAOS on TI was substantial (βtotal = 0.5000, SE = 0.0554, *p* < 0.001), the direct effect (βdirect = 0.2232, SE = 0.0583, *p* = 0.2576) was still significant, while all three mediators remained significant predictors (Figure 3).

Overall, the three mediators partially mediated the relationship between PAOS and TI (IE overall = 0.2768, SE = 0.0426, 95% CI: LL = 0.2010 to UL = 0.3684), indicating that employees with low scores on occupational safety and health perception OSHP and perceived organizational effectiveness POE, and high scores on perceived job insecurity PJI, were more likely to have higher levels of TI turnover intentions. In light of this research, occupational safety and health positive perception, positively perceived organizational effectiveness and a low perception regarding job insecurity significantly impact hospitality industry staff turnover intentions.

The findings of this research indicate crucial elements influencing the turnover intentions of Romanian hospitality industry personnel during the COVID pandemic. This research revealed that pandemic accentuated occupational stress (PAOS) and perceived job instability (PJI) have positive impacts on employees’ turnover intention (TI), but occupational safety and health perception (OSHP) and perceived organizational effectiveness (POE) have negative effects (TI). Moreover, this study found a significant partial parallel mediation of occupational safety and health perception, perceived organizational effectiveness and perceived job insecurity over the relationship between pandemic accentuated occupational stress on hospitality industry staff turnover intentions.

The results demonstrated that, particularly during the COVID crisis, assistance provided to employees by organizations, in terms of appealing occupational safety and health measures and perceived effectiveness strengthened employees’ commitment and significantly lowered their intentions to leave the organization. On the other hand, if the business did not address pandemic-induced occupational stress and employee perceptions of job insecurity, the possibility of employees leaving was imminent.

The findings of the study provide theoretical and practical advances to lowering the detrimental impact of perceived job instability and pandemic-exacerbated workplace stress on turnover intentions, both of which have been identified as major drivers of occupational strain.

The primary limitation of our research comes from the characteristics of cross-sectional studies, namely that the temporal link between the outcome of hospitality industry staff turnover intentions and the exposure to pandemic accentuated occupational stress, could not be determined because both were examined at the same time.

Without longitudinal data, it is hard to establish a true cause and effect link. Our study factors, that were statistically different during the assessment period, may be a result of a plethora of other unanticipated variables, rather than a cause. As a result, predicting outcomes based on these variations is challenging. Additional data from earlier time points before the actual evaluation, or data collected over a longer period of time, might assist in elucidating the elements that influence hospitality sector worker turnover intentions.

Another limitation of this research is represented by the fact that only two items were used to measure the perceived organizational effectiveness and perceived job insecurity constructs and, therefore, the complexity of these two constructs may not have been fully assessed.

Future studies may identify different antecedents that predict hospitality industry staff turnover intentions. Besides, other parallel or sequential mediators of the relationship between pandemic accentuated occupational stress and hospitality industry staff turnover intentions, apart from occupational safety and health perception, perceived organizational effectiveness and perceived job insecurity, could be suggested. New directions for future research could be designed to focus on talent management directly improving sustainable performance [110], enhancing the motivation of employees [111], taking an in-depth look at individual behaviors [112] and correlating salary, happiness, and life satisfaction, for a better perspective on the factors known to hinder turnover intentions [113].

While this study was limited to Romania, expanding the study to other Eastern European nations is another prospective future research topic that might allow for comparisons with the present study findings.

## Figures and Tables

**Table 1:** Correlations between the scale’s 5 factors and turnover intentions.

	Factor 3	Factor 1	Factor 4	Factor 5	Factor 2	Turnover Intentions
Factor 3—COVID fear	—					
Factor 1—Occupational safety and health perception	0.733 ***	—				
Factor 4—Perceived organizational effectiveness	0.354 ***	0.556 ***	—			
Factor 5—Perceived job insecurity	−0.184 ***	−0.201 ***	−0.202 ***	—		
Factor 2—Pandemic accentuated occupational stress	−0.32 ***	−0.447 ***	−0.354 ***	0.352 ***	—	
Turnover intentions	−0.366 ***	−0.524 ***	−0.438 ***	0.371 ***	0.454 ***	—
Means	3.55	4.03	4.25	2.98	2.08	2.28
Standard deviations	1.32	0.983	1.03	1.3	1.3	1.44

*** significant at *p* < 0.001.

**Table 2:** Centrality measures for the items associated with the 5 factors.

Variable	Network			
	Betweenness	Closeness	Strength	Expected Influence
item 1	0.569	1.107	0.144	−0.682
item 2	0.086	0.717	−0.833	0.163
item 3	−1.364	−1.439	−1.784	−0.407
item 4	0.730	0.247	0.202	0.263
item 5	2.341	0.874	0.428	−1.472
item 6	−0.397	0.201	−0.608	−0.145
item 7	0.569	1.050	1.800	1.385
item 8	−1.203	1.082	1.417	1.512
item 9	0.569	1.322	−0.027	6.192 × 10^−4^
item 10	−0.236	−0.908	−1.583	−2.053
item 11	0.569	−0.698	0.901	1.203
item 12	−1.364	−1.653	−0.556	0.329
item 13	0.408	−0.767	0.769	0.798
item 14	−0.236	−0.450	−0.078	−0.248
item 15	−1.041	−0.685	−0.191	−0.645

**Table 3:** Confirmatory factor analysis, factor loadings.

	Factor					
	1	2	3	4	5	Uniqueness
Item 1				0.969		0.0025
Item 2				0.449		0.47384
Item 3		0.781				0.4179
Item 4		0.654				0.36872
Item 5		1.041				−0.07253
Item 6	0.541					0.40795
Item 7	0.817					0.19177
Item 8	0.92					0.12323
Item 9	0.689					0.38457
Item 10	0.49					0.60201
Item 11			0.651			0.22273
Item 12			0.884			0.23582
Item 13			0.872			0.14506
Item 14					0.731	0.44878
Item 15					0.656	0.54335

Note. ‘Minimum residual’ extraction method was used in combination with a ‘oblimin’ rotation.

**Table 4:** Model coefficients of OSHP, POE PJI, PAOS and CF predicting TI.

			95% Confidence Interval				
Predictor	Estimate	SE	Lower	Upper	t	*p*	Stand. Estimate
Intercept	3.9724	0.4812	3.0256	4.9191	8.256	<0.001	
Factor 3	0.0252	0.0741	−0.1206	0.171	0.34	0.734	0.022
Factor 1	−0.45	0.119	−0.6841	−0.2159	−3.782	<0.001	−0.29
Factor 4	−0.2377	0.0793	−0.3937	−0.0817	−2.997	0.003	−0.1667
Factor 5	0.1963	0.056	0.0861	0.3065	3.505	<0.001	0.1725
Factor 2	0.223	0.0584	0.1081	0.338	3.818	<0.001	0.2026

## Data Availability

Not applicable.
